# Supplementary figures and images for: Whole-genome sequencing revealed genetic diversity and selection of Guangxi indigenous chickens
Source: PLoS One. 2022 Mar 15;17(3):e0250392. doi: 10.1371/journal.pone.0250392 (PMC8923445; doi:10.1371/journal.pone.0250392)

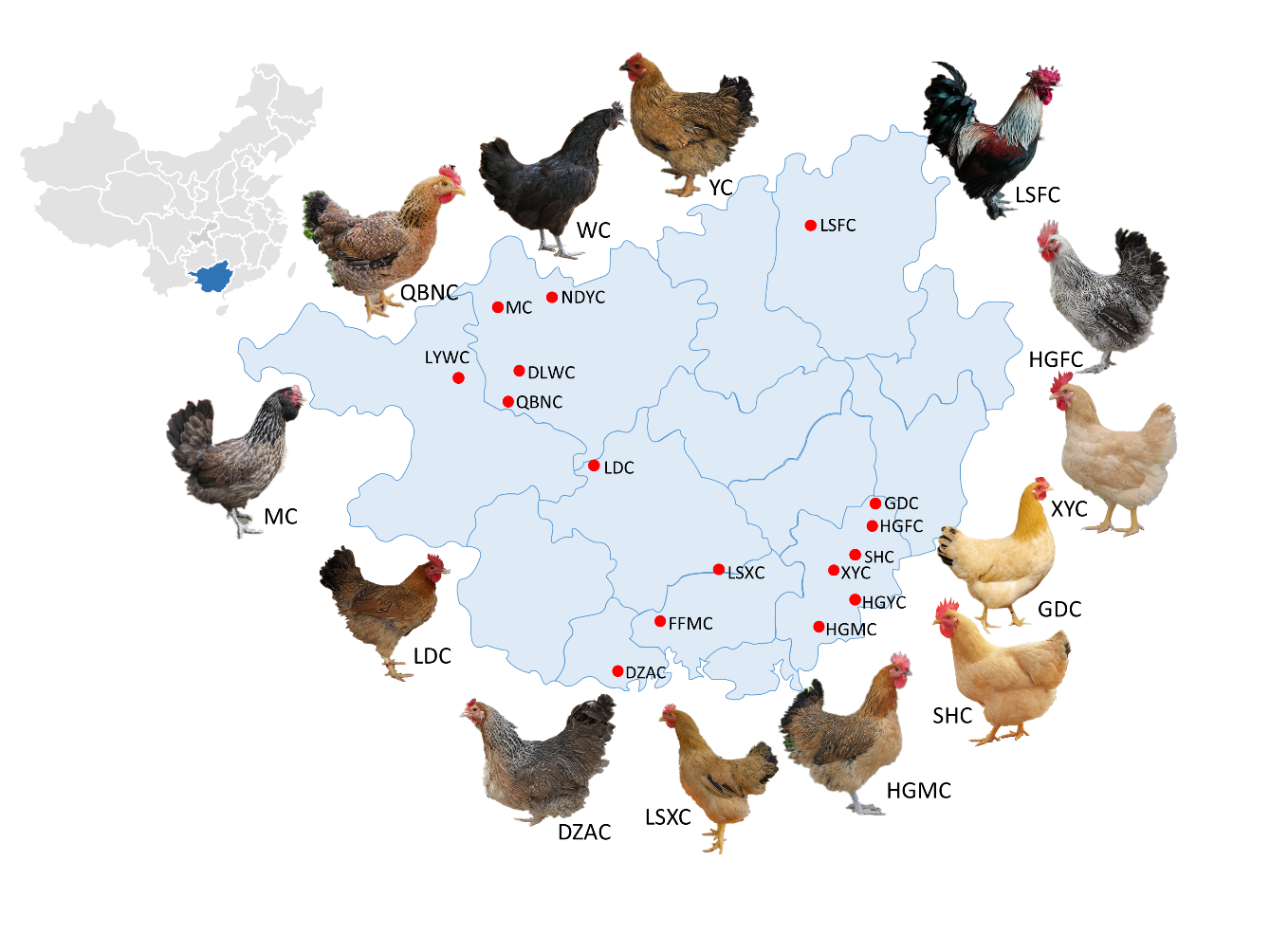


**S1 Fig. Geographic distribution and appearances of typical female chickens.**

Supplement: S1 Fig — (DOCX) [file pone.0250392.s001.docx]

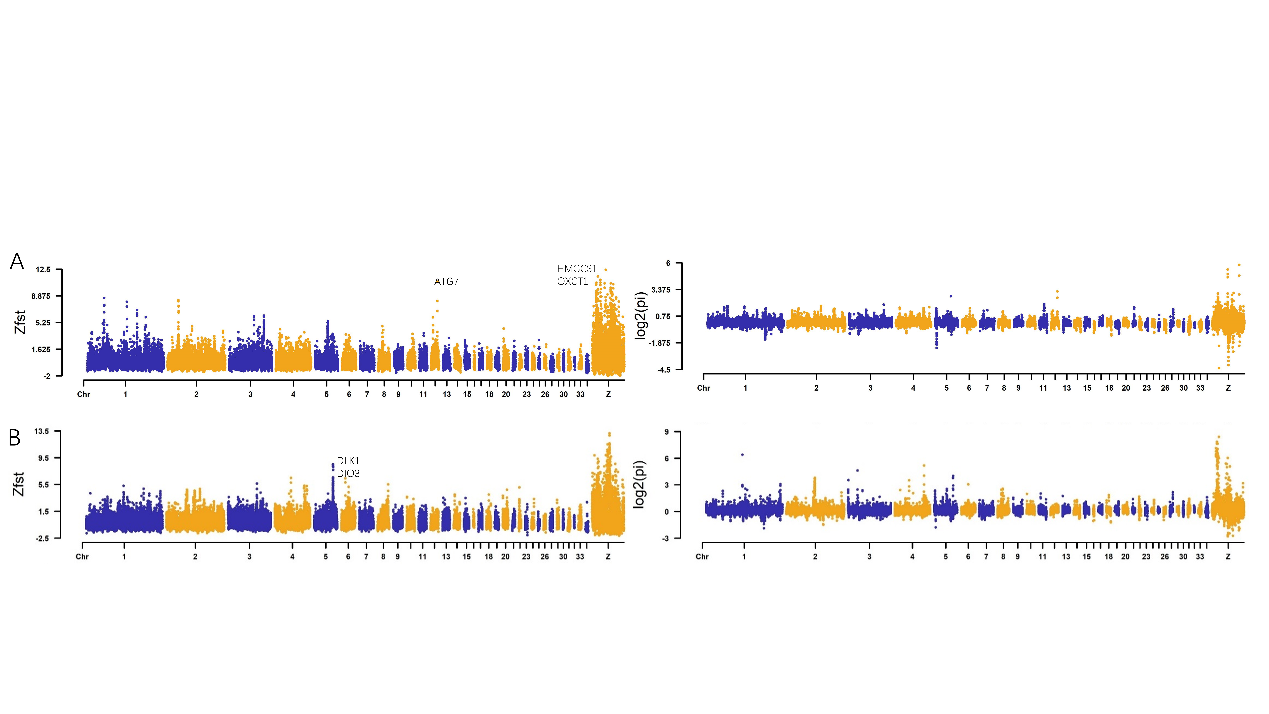


**S6 Fig. ZFst values and Log 2 (pi).** (A) XYC and SHC. (B) GDC and SHC.

Supplement: S6 Fig — (A) XYC and SHC. (B) GDC and SHC. (DOCX) [file pone.0250392.s006.docx]
