# Supplementary material for: Whole-genome sequencing revealed genetic diversity and selection of Guangxi indigenous chickens
Source: PLoS One. 2022 Mar 15;17(3):e0250392. doi: 10.1371/journal.pone.0250392 (PMC8923445; doi:10.1371/journal.pone.0250392)
Supplement: S2 Fig — (DOCX) [file pone.0250392.s002.docx]

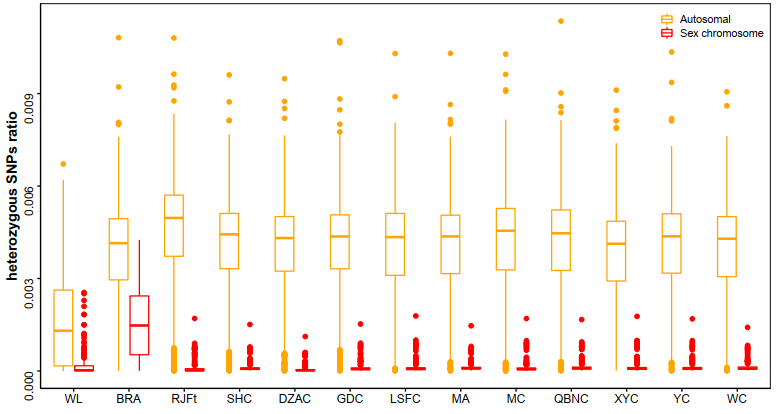


**S2 Fig.** **Boxplot showing heterozygous SNP rate of autosomes (left) and Z chromosome (right) between each chicken population.**
