# Supplementary material for: Whole-genome sequencing revealed genetic diversity and selection of Guangxi indigenous chickens
Source: PLoS One. 2022 Mar 15;17(3):e0250392. doi: 10.1371/journal.pone.0250392 (PMC8923445; doi:10.1371/journal.pone.0250392)
Supplement: S3 Fig — (A) Distribution of isochron along chromosome. (B) Chromosome length and gene density. (C) Scatter plot of Indel number and GC content in isochrones per 100kb window. (D) Distribution of ROH in chicken breeds. (DOCX) [file pone.0250392.s003.docx]

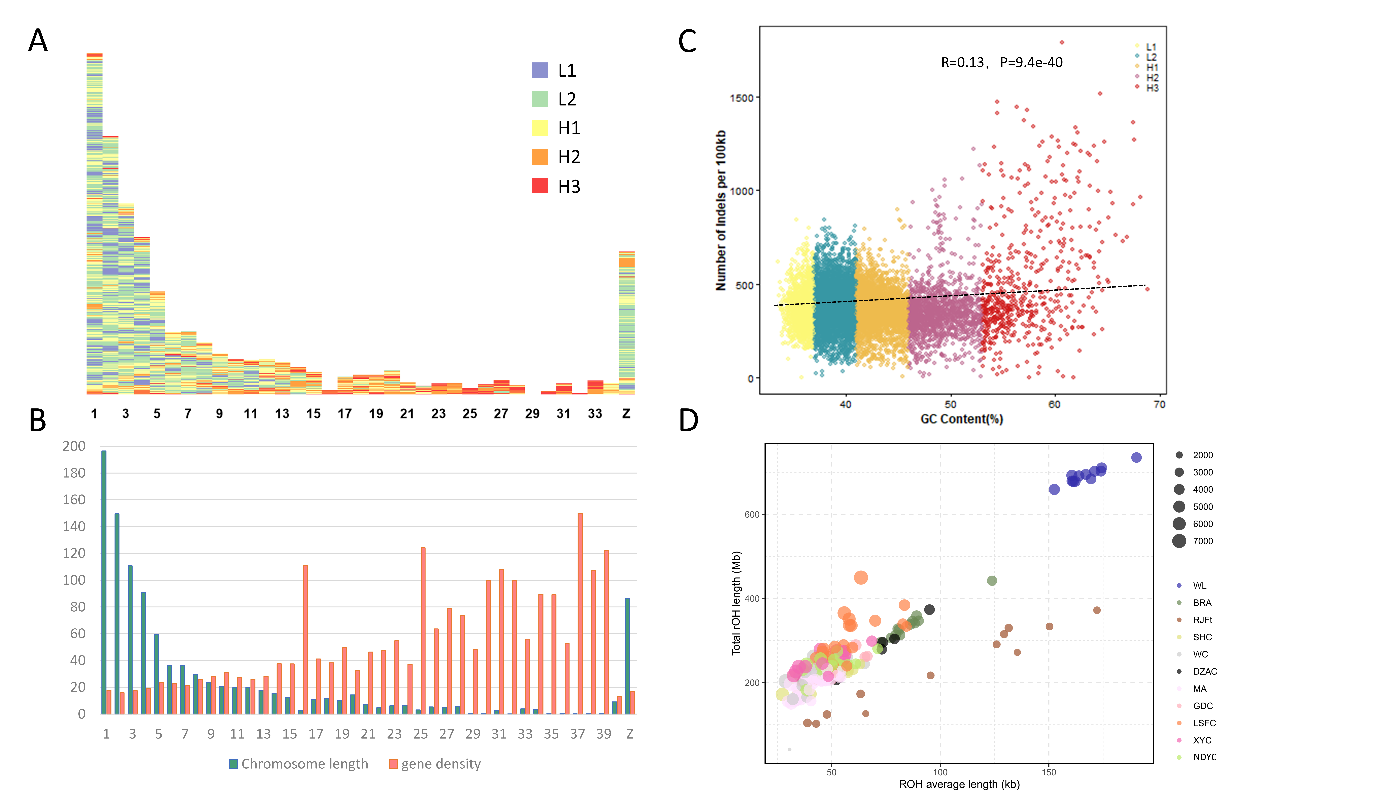


**S3 Fig. Isochores and ROH distribution of Guangxi chicken.** (A) Distribution of isochron along chromosome. (B) Chromosome length and gene density. (C) Scatter plot of Indel number and GC content in isochrones per 100kb window. (D) Distribution of ROH in chicken breeds.
