# Supplementary material for: Whole-genome sequencing revealed genetic diversity and selection of Guangxi indigenous chickens
Source: PLoS One. 2022 Mar 15;17(3):e0250392. doi: 10.1371/journal.pone.0250392 (PMC8923445; doi:10.1371/journal.pone.0250392)
Supplement: S4 Fig — Each population separated by white dotted line. (DOCX) [file pone.0250392.s004.docx]

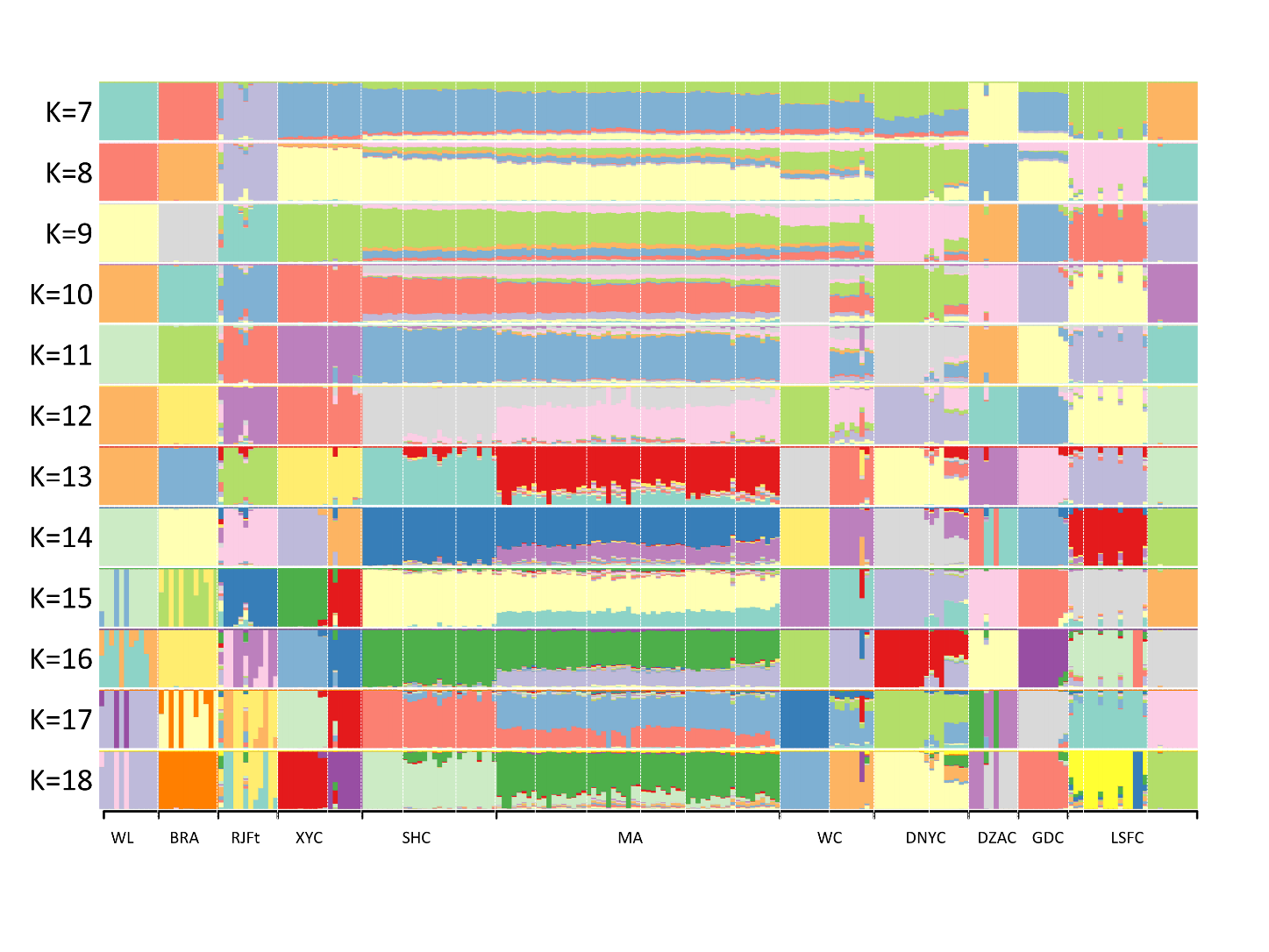


**S4 Fig.** **Admixture analysis with K values running from 7 to 18.** Each population separated by white dotted line.
