# Supplementary material for: Whole-genome sequencing revealed genetic diversity and selection of Guangxi indigenous chickens
Source: PLoS One. 2022 Mar 15;17(3):e0250392. doi: 10.1371/journal.pone.0250392 (PMC8923445; doi:10.1371/journal.pone.0250392)
Supplement: S5 Fig — The row represents the SNP position and the column represents the individual. Light blue denotes reference alleles while red indicates alternative homozygous alleles, yellow means heterozygous and dark blue means missing. (DOCX) [file pone.0250392.s005.docx]

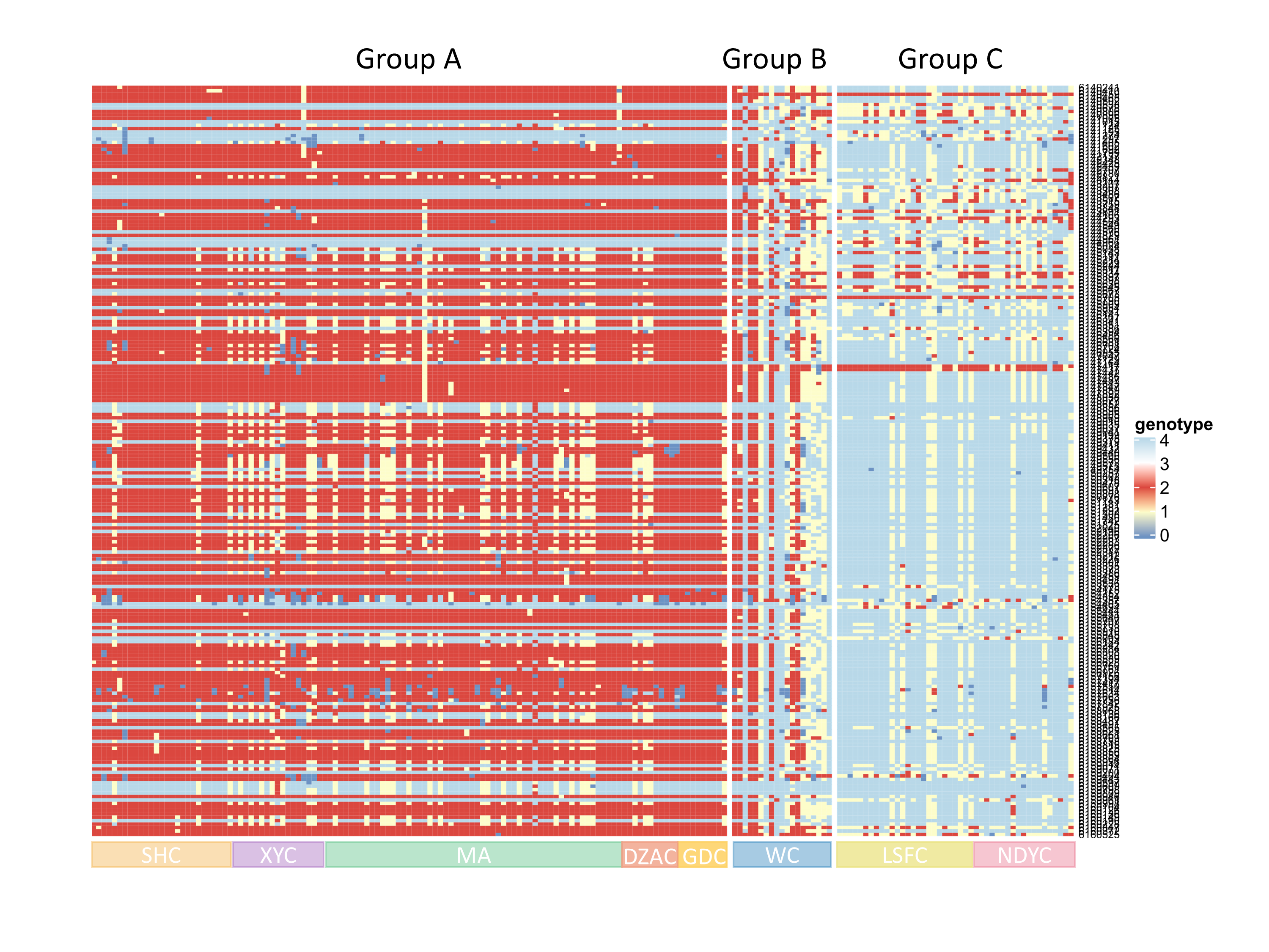


**S5 Fig. The genotype of fixed SNPs in chr24: 6.14Mb~6.18Mb of individuals.** The row represents the SNP position and the column represents the individual. Light blue denotes reference alleles while red indicates alternative homozygous alleles, yellow means heterozygous and dark blue means missing.
