# Supplementary material for: Whole-genome sequencing revealed genetic diversity and selection of Guangxi indigenous chickens
Source: PLoS One. 2022 Mar 15;17(3):e0250392. doi: 10.1371/journal.pone.0250392 (PMC8923445; doi:10.1371/journal.pone.0250392)
Supplement: Attachment — Submitted filename: Response to Reviewers-2.docx [file pone.0250392.s021.docx]

Dear Chiang and dear editors,

Re: Manuscript ID: PONE-D-21-10366R1

Title: Whole genome sequencing revealed genetic diversity and selection of Guangxi indigenous chickens

Thank you for your letter and for the reviewers and Staff editor’s comments. The point to the point response to the reviewer's comments are listed as following: Revisions in the text are shown using the yellow highlight for additions and strikethrough font ~~[example]~~ for deletions.

Journal Requirements:

Please review your reference list to ensure that it is complete and correct. If you have cited papers that have been retracted, please include the rationale for doing so in the manuscript text, or remove these references and replace them with relevant current references. Any changes to the reference list should be mentioned in the rebuttal letter that accompanies your revised manuscript. If you need to cite a retracted article, indicate the article’s retracted status in the References list and also include a citation and full reference for the retraction notice.

**Response:** We re-examined the references list to make sure that the information was complete and correct and no papers have been retracted.

**Staff Editor Comments**
1. For editorial purposes please include a legend on the Y-axis for figure 1A. The legend text indicates that this represents SNP and Indel number, but the scale goes from 0-0.16.

[Note: HTML markup is below. Please do not edit.]

**Response:** Thank you your valuable advice. We have modified figure 1A with a Y-axis legend renamed ‘PACE-FIG 1’ on the submission website. I also attached the figure below.

Fig1. The number, distribution, and GC content of SNP and INDEL.

Reviewers' comments:
Reviewer's Responses to Questions

**Comments to the Author**

1. If the authors have adequately addressed your comments raised in a previous round of review and you feel that this manuscript is now acceptable for publication, you may indicate that here to bypass the “Comments to the Author” section, enter your conflict of interest statement in the “Confidential to Editor” section, and submit your "Accept" recommendation.

Reviewer #2: (No Response)

**Response:** Thank you.

2. Is the manuscript technically sound, and do the data support the conclusions?

The manuscript must describe a technically sound piece of scientific research with data that supports the conclusions. Experiments must have been conducted rigorously, with appropriate controls, replication, and sample sizes. The conclusions must be drawn appropriately based on the data presented.

Reviewer #2: Yes

**Response:** Thank you.

  3. Has the statistical analysis been performed appropriately and rigorously?

Reviewer #2: Yes

**Response:** Thank you.

  4. Have the authors made all data underlying the findings in their manuscript fully available?

The [PLOS Data policy](https://apc01.safelinks.protection.outlook.com/?url=http%3A%2F%2Fwww.plosone.org%2Fstatic%2Fpolicies.action%23sharing&data=04%7C01%7Cyangmanman%40genomics.cn%7C331cba98018c4836f53108d9c6aa92c7%7C853aa2281adc4d91bb286065c1e9963d%7C0%7C1%7C637759258234614586%7CUnknown%7CTWFpbGZsb3d8eyJWIjoiMC4wLjAwMDAiLCJQIjoiV2luMzIiLCJBTiI6Ik1haWwiLCJXVCI6Mn0%3D%7C2000&sdata=hLLEEjwFDcfXybKhdWWm3JyTs6Zlo5MCbWP0%2BoctoDU%3D&reserved=0) requires authors to make all data underlying the findings described in their manuscript fully available without restriction, with rare exception (please refer to the Data Availability Statement in the manuscript PDF file). The data should be provided as part of the manuscript or its supporting information, or deposited to a public repository. For example, in addition to summary statistics, the data points behind means, medians and variance measures should be available. If there are restrictions on publicly sharing data—e.g. participant privacy or use of data from a third party—those must be specified.

Reviewer #2: Yes

**Response:** Thank you.

  5. Is the manuscript presented in an intelligible fashion and written in standard English?

PLOS ONE does not copyedit accepted manuscripts, so the language in submitted articles must be clear, correct, and unambiguous. Any typographical or grammatical errors should be corrected at revision, so please note any specific errors here.

Reviewer #2: Yes

**Response:** Thank you.

  6. Review Comments to the Author

Please use the space provided to explain your answers to the questions above. You may also include additional comments for the author, including concerns about dual publication, research ethics, or publication ethics. (Please upload your review as an attachment if it exceeds 20,000 characters)

Reviewer #2: (No Response)

**Response:** Thank you.

  7. PLOS authors have the option to publish the peer review history of their article ([what does this mean?](https://apc01.safelinks.protection.outlook.com/?url=https%3A%2F%2Fjournals.plos.org%2Fplosone%2Fs%2Feditorial-and-peer-review-process%23loc-peer-review-history&data=04%7C01%7Cyangmanman%40genomics.cn%7C331cba98018c4836f53108d9c6aa92c7%7C853aa2281adc4d91bb286065c1e9963d%7C0%7C1%7C637759258234614586%7CUnknown%7CTWFpbGZsb3d8eyJWIjoiMC4wLjAwMDAiLCJQIjoiV2luMzIiLCJBTiI6Ik1haWwiLCJXVCI6Mn0%3D%7C2000&sdata=dWkEI8WXpz8QJYff3s2uN3lsA7AMlcZq%2Bgu47sBvV%2FA%3D&reserved=0)). If published, this will include your full peer review and any attached files.


If you choose “no”, your identity will remain anonymous but your review may still be made public.


**Do you want your identity to be public for this peer review?** For information about this choice, including consent withdrawal, please see our [Privacy Policy](https://apc01.safelinks.protection.outlook.com/?url=https%3A%2F%2Fwww.plos.org%2Fprivacy-policy&data=04%7C01%7Cyangmanman%40genomics.cn%7C331cba98018c4836f53108d9c6aa92c7%7C853aa2281adc4d91bb286065c1e9963d%7C0%7C1%7C637759258234614586%7CUnknown%7CTWFpbGZsb3d8eyJWIjoiMC4wLjAwMDAiLCJQIjoiV2luMzIiLCJBTiI6Ik1haWwiLCJXVCI6Mn0%3D%7C2000&sdata=qwCnNFB7a8j%2F2IAzWOB6vGb7K0s6l%2FjBK5gQBC1gr7o%3D&reserved=0).

Reviewer #2: No

[NOTE: If reviewer comments were submitted as an attachment file, they will be attached to this email and accessible via the submission site. Please log into your account, locate the manuscript record, and check for the action link "View Attachments". If this link does not appear, there are no attachment files.]

While revising your submission, please upload your figure files to the Preflight Analysis and Conversion Engine (PACE) digital diagnostic tool, [https://pacev2.apexcovantage.com/](https://apc01.safelinks.protection.outlook.com/?url=https%3A%2F%2Fpacev2.apexcovantage.com%2F&data=04%7C01%7Cyangmanman%40genomics.cn%7C331cba98018c4836f53108d9c6aa92c7%7C853aa2281adc4d91bb286065c1e9963d%7C0%7C1%7C637759258234614586%7CUnknown%7CTWFpbGZsb3d8eyJWIjoiMC4wLjAwMDAiLCJQIjoiV2luMzIiLCJBTiI6Ik1haWwiLCJXVCI6Mn0%3D%7C2000&sdata=k5WzQsIC%2F3QkWyVEDcrnfdCtIBD7x6x1UHuP37XfoDY%3D&reserved=0). PACE helps ensure that figures meet PLOS requirements. To use PACE, you must first register as a user. Registration is free. Then, login and navigate to the UPLOAD tab, where you will find detailed instructions on how to use the tool. If you encounter any issues or have any questions when using PACE, please email PLOS at [figures@plos.org](mailto:figures@plos.org). Please note that Supporting Information files do not need this step.

**Response:** The PACE corrected figures has been uploaded to the submission website and renamed as PACE-FIG1~4.
